# A Case of Hidden Synchronous Colorectal Cancer

**DOI:** 10.7759/cureus.117746

**Published:** 2026-10-10

**Authors:** Amanda Eshak, Spiros Kavvathas, Rachel Sergah, Harold Paredes

**Affiliations:** 1 Internal Medicine, Arrowhead Regional Medical Center, Colton, USA; 2 Gastroenterology, Arrowhead Regional Medical Center, Colton, USA

**Keywords:** apple core lesion, braf mutation, chromosomal instability, microsatellite instability, synchronous colorectal cancer

## Abstract

Synchronous colorectal cancer (SCC) is uncommon and refers to multiple primary colorectal cancers arising simultaneously. The following case presents the clinical course of a 54-year-old female patient who presented with abdominal pain. She was found to have a mass in the hepatic flexure and another mass in the splenic flexure, both of which were found to be invasive adenocarcinoma and negative for mismatch repair (MMR) deficiency on the testing performed. Clinicians should be aware of SCC, as colonoscopy can identify tumors not visualized on computed tomography (CT), and early surgical intervention can ultimately be curative for patients.

## Introduction

Synchronous colorectal cancer (SCC) is an uncommon form of colorectal malignancy referring to at least two primary malignancies occurring simultaneously in the colon or rectum. These can be collectively diagnosed as SCC at the same time or within one year of the first mass’ diagnosis. Synchronous colon cancers are typically treated with two separate resections or one extended resection. The prevalence of the disease is typically 3.5% of all colorectal cancers, with a male-to-female predominance of 1.8:1 [1].

Patients at increased risk of developing synchronous colorectal carcinomas are those with histories of inflammatory bowel diseases such as Crohn’s disease and ulcerative colitis. Other conditions that can increase risk include hereditary nonpolyposis colon cancer (Lynch syndrome), familial adenomatous polyposis (FAP), presence of serrated polyps, and hyperplastic polyposis [1]. Studies have shown that chromosomal instability (CIN), microsatellite instability (MSI), and genetic methylation have also been associated with an increased risk of developing SCC [2].

Some studies suggest that these forms of synchronous malignancies may carry a higher mortality as compared to single solitary tumors [3]. Of note, synchronous tumors have been predisposed to be typically located in the same intestinal segment [4]. In our patient, the tumors were at the hepatic and splenic flexures, and computed tomography (CT) imaging identified only one lesion, which was localized differently from the colonoscopy findings.

## Case presentation

The patient is a 54-year-old female who presented to the hospital due to progressively worsening abdominal pain associated with nausea and vomiting over a three-day period. She reported a history of intermittent, diffuse, generalized abdominal pain of three months’ duration and noted an unintentional nine-pound weight loss in the two weeks prior to admission. She also reported no bowel movements or passing gas over the three-day period. Her past medical history was significant for gastritis and previously treated *Helicobacter pylori* infection. She denied a family history of colorectal, uterine, or ovarian cancer. Initial vital signs included a pulse rate of 91, respiratory rate of 16, blood pressure of 123/70 mmHg, and oxygen saturation of 97% on room air. Physical examination revealed a moderately distended abdomen with mild tenderness to palpation in all four quadrants.

A CT scan with IV contrast of the abdomen and pelvis was obtained, which revealed diffuse dilation of the small bowel and right hemicolon secondary to a focal stricture and apple core lesion in the distal transverse colon (Figures 1A-1B). These findings were concerning for a large bowel obstruction secondary to a colonic mass, with high suspicion for malignancy. A nasogastric tube was inserted and set to suction to relieve intestinal obstruction; however, the patient had bowel movements and was passing gas shortly after admission. Given that the obstruction was likely relieved, she was then administered 4 L of polyethylene glycol in preparation for a colonoscopy to further investigate the mass. 

**Figure 1:**
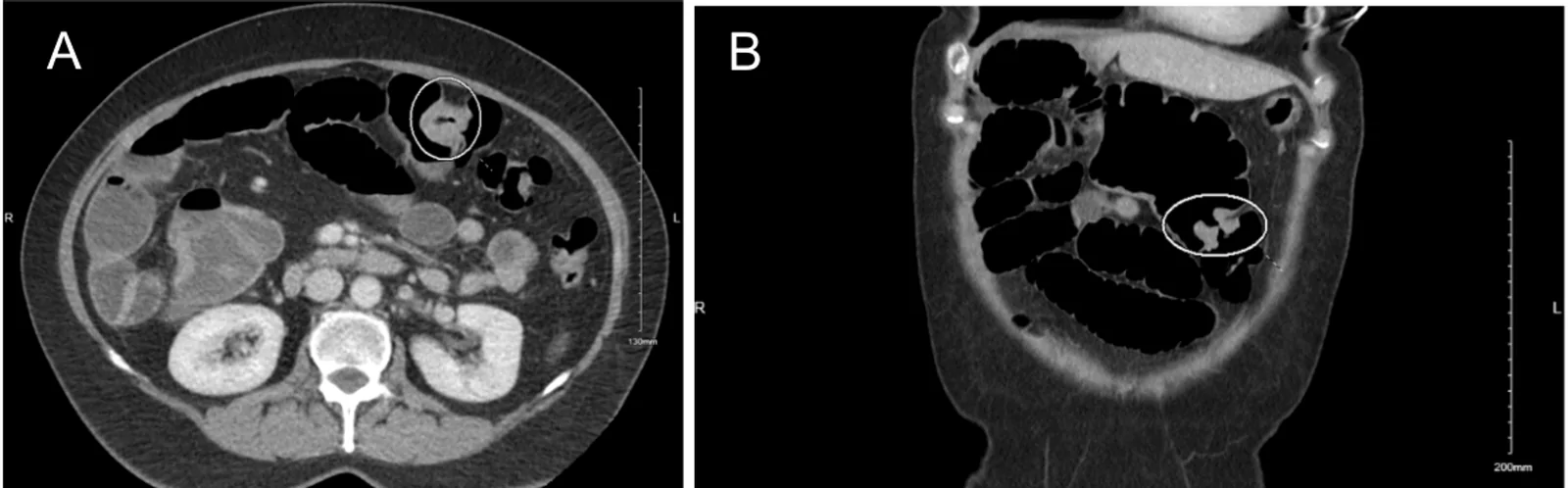
Computed tomography (CT) scan of the abdomen and pelvis. (A) Axial CT scan of the abdomen and pelvis with IV contrast, revealing diffuse dilation of the small bowel and right hemicolon secondary to a focal stricture and apple core lesion in the distal transverse colon. (B) Coronal CT scan of the abdomen and pelvis with IV contrast, revealing diffuse dilation of the small bowel and right hemicolon secondary to a focal stricture and apple core lesion in the distal transverse colon.

The subsequent day, a colonoscopy was performed, revealing a circumferential, ulcerated mass at the hepatic flexure of the colon (Figures 2A-2B) and a half-circumferential, polypoid-shaped mass at the splenic flexure of the colon (Figures 3A-3B). The ulcerated mass at the hepatic flexure was unable to be traversed with the colonoscope. The mass in the splenic flexure was injected with Eleview®, Medtronic Parkway, Minneapolis, MN, USA, a submucosal injectable composition used in the lifting of polyps, and demonstrated no lifting. This finding indicated that the lesion was not only unresectable via colonoscopy but also indicated possible tissue invasion by the mass. Multiple biopsies were taken of both masses using cold forceps and sent for pathological examination.

**Figure 2:**
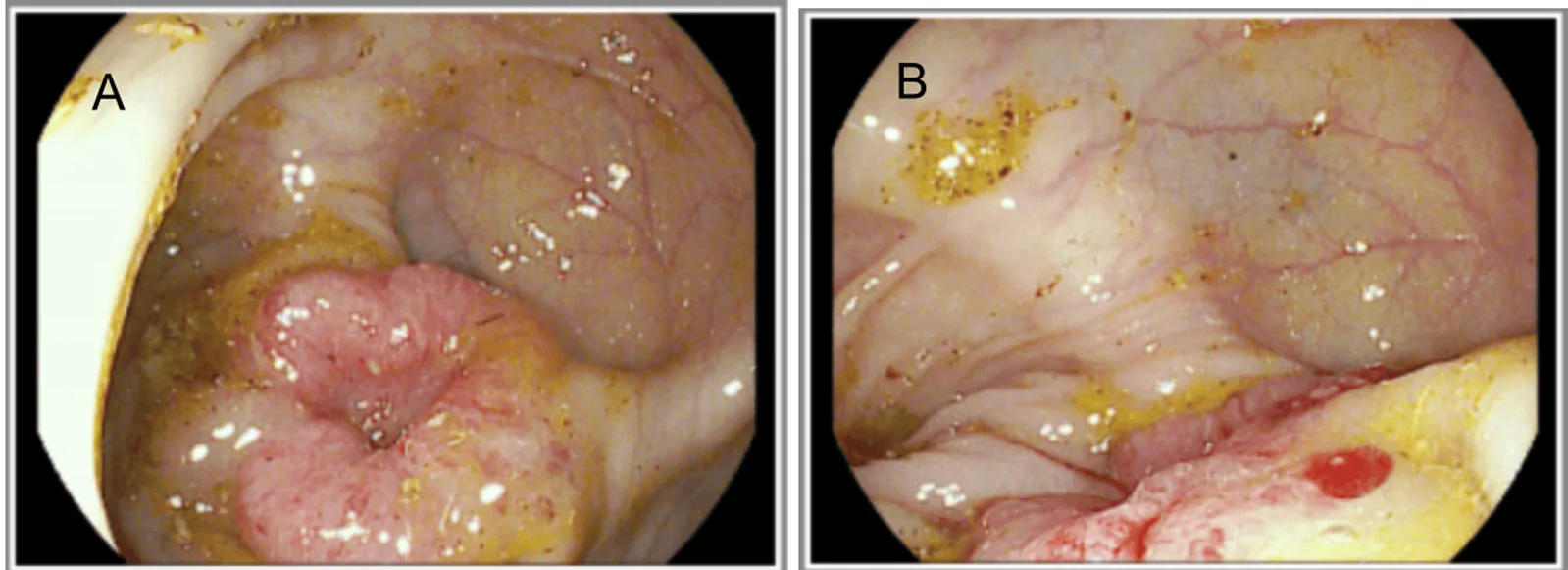
(A) Circumferential, ulcerated mass at the hepatic flexure of the colon visualized by colonoscopy. (B) Additional view of the hepatic flexure mass.

**Figure 3:**
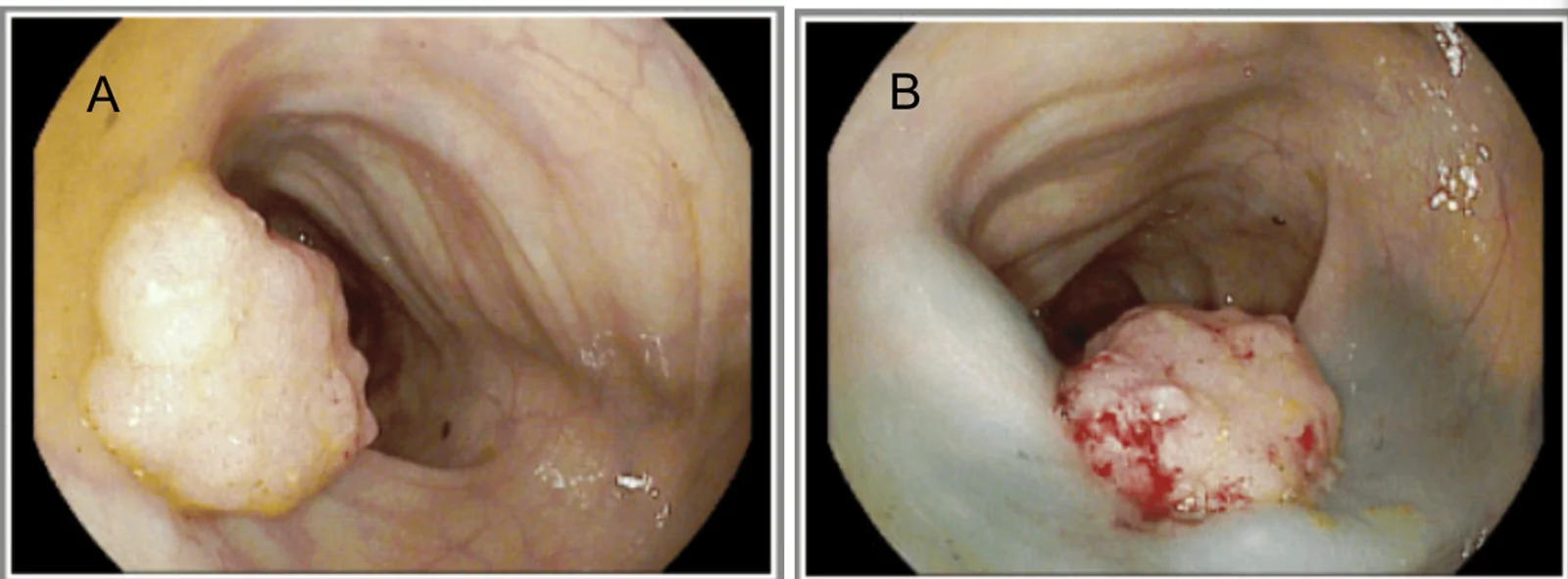
(A) Half-circumferential, polypoid-shaped mass at the splenic flexure of the colon visualized by colonoscopy. (B) Additional view of the splenic flexure lesion.

The patient’s serum carcinoembryonic antigen (CEA) and carbohydrate antigen 19-9 (CA 19-9) were found to be elevated at 22.9 ng/mL (normal 0-3) and 88 U/mL (normal <34), respectively (Table 1). A CT of the chest with intravenous contrast was also performed, showing no evidence of metastatic disease within the thorax. Head and neck imaging was not pursued due to lack of symptoms that would suggest metastatic spread to these regions. Pathology reports of biopsies revealed a well-differentiated invasive adenocarcinoma at the hepatic flexure of the colon and a complex tubulovillous adenoma with severe dysplasia at the splenic flexure. Both samples were negative for deficiency in mismatch repair (MMR) proteins and therefore did not receive reflex testing for MSI or BRAF mutations. 

**Table 1:** Tumor marker results at initial presentation.

Laboratory parameter	Patient value	Reference range/normal value
Carcinoembryonic antigen (CEA)	22.9 ng/mL	0-3 ng/mL
Carbohydrate antigen 19-9 (CA 19-9)	88 U/mL	<34 U/mL

Subtotal colectomy with ileosigmoid anastomosis and lymphadenectomy were performed, with negative margins for carcinoma confirmed by pathologic study. The hepatic flexure mass measured approximately 5 cm × 3 cm and, on further study, revealed moderately differentiated invasive colonic adenocarcinoma involving the mucosa, submucosa, and muscularis propria. The splenic flexure mass measured 5 cm × 2.5 cm × 0.5 cm and revealed moderately differentiated invasive colonic adenocarcinoma extending through the muscularis propria with microscopic involvement of the serosa; however, no invasion through the serosa was noted. All 14 resected lymph nodes were negative for metastatic carcinoma. The final TNM staging yielded T4aN0M0.

The patient was postoperatively administered cefoxitin, 2 g every eight hours intravenously for 48 hours total, and maintained on a clear liquid diet with later removal of the NG tube, as she began to tolerate an oral diet. The postoperative course was complicated by non-bloody diarrhea, which was resolved with banana flakes (transgalactooligosaccharides), and the patient was discharged on postoperative day 8 in stable condition.

## Discussion

Synchronous colorectal carcinoma is an uncommon condition in which two or more primary colorectal carcinomas are concomitantly discovered in a patient [3]. The incidence rate has most notably been demonstrated by Lam et al. in a systematic review of 39 studies, which involved over 100,000 colorectal cancer patients. They were able to clarify a synchronous colorectal carcinoma incidence rate of 3.5% of all colorectal carcinomas [1]. There is a slightly higher incidence rate of synchronous colorectal carcinoma in males, with a ratio of 1.8:1 noted by Lam et al., and a higher likelihood of right-sided colonic involvement when compared to those of solitary colorectal carcinomas [1,5]. In previous studies, prognosis was thought to be identical between synchronous and solitary colorectal carcinomas; however, Nosho et al. were able to perform a prospective study on the topic, which suggested that SCCs could have a worse prognosis compared to solitary colorectal cancers [3,6].

The pathophysiology of synchronous colorectal carcinoma may be explained by the process of cellular damage that induces carcinogenicity. Damage at the molecular level can cause defects in DNA MMR and lead to MSI within the DNA of cells. This, in turn, may lead to activation of oncogenes, such as BRAF, and inhibition of tumor suppressor genes through methylation, with methylation products known as CpG island methylator phenotype (CIMP) [3,5]. When compared to solitary colorectal carcinomas, synchronous colorectal carcinomas were noted to have more BRAF mutations and higher states of MSI and CIMP, all of which could influence more aggressive growth patterns [3]. Furthermore, it is also suggested that synchronous colorectal carcinomas experience these effects through a shared “field effect,” which has been further refined into the “etiological field effect.” This etiological field effect provides a model for the induction of carcinogenicity by hypothesizing that etiological exposures create microenvironmental changes that generate a shared pathway for different tumors to grow [6]. However, in the case presented above, the patient’s tumors did not show a loss of DNA MMR protein expression on histopathological testing. This does not exclude other genetic or epigenetic alterations that were not tested. 

This case is notable because CT imaging demonstrated only one lesion in the distal transverse colon, while colonoscopy found two tumors at the hepatic and splenic flexures. Many of the current case reports evaluating synchronous colorectal carcinomas involve patients already presenting with advanced disease and metastasis, most notably to the kidneys and throughout the gastrointestinal tract [7-10]. In our patient, imaging showed no distant metastases. Given the initial vague abdominal symptoms of the patient, potential differential diagnoses included pancreatitis, appendicitis, and small bowel obstruction. Discovery of a potential mass was not made until a CT abdomen with IV contrast was performed, which only visualized one of the masses, followed by a colonoscopy, which then visualized both masses. This furthers the importance of colonoscopy in the diagnosis of colon cancer, as it is not only superior to imaging for detecting multiple lesions but is also superior to imaging for localizing and tattooing lesions for later surgical resection. In the above case, the patient reported never having a colonoscopy or esophagogastroduodenoscopy (EGD) in the past. The current recommendations of the US Preventive Services Task Force for colorectal cancer screening are to begin colonoscopy screening at 45 years of age and to repeat screening at time intervals indicated by the initial results. Unfortunately, a recent study has shown that patient adherence to these recommendations is only 59% thus far in 2023 [11]. Our case highlights the presentation of this uncommon colorectal disease and emphasizes the importance of colonoscopic evaluation in identifying synchronous lesions. 

Per the National Comprehensive Cancer Network (NCCN) guideline recommendations, initial studies for suspected colorectal malignancy include chest, abdomen, and pelvis CT imaging with intravenous or oral contrast for staging. The lesions in question also require further investigation via colonoscopy with biopsy. Biopsied specimens should be histologically tested for MSI, which is caused by deficiency of DNA MMR. If the lesions are resectable, patients should also have a complete blood count (CBC), chemistry profile, and CEA level evaluated prior to surgery [12].

Treatment varies depending on the TNM staging of the lesions but ultimately revolves around surgical resection. For non-metastatic disease, resection of affected tissue is recommended alongside en bloc removal of at least 12 regional lymph nodes [12]. Interestingly, Lee et al. demonstrated no difference in complication rate between those undergoing extensive colectomy and those receiving two regional resections; however, they did note a benefit in decreased frequency of bowel movements for the two regional resections group when compared to the colectomy group [13]. 

## Conclusions

SCC is an uncommon form of colorectal cancer and can be identified on colonoscopy. Clinicians should be aware of this disease entity and proceed with prompt surgical evaluation for resection if detected. This case is notable in that two separate invasive adenocarcinomas were identified at the hepatic and splenic flexures; CT imaging revealed only one mass, and both tumors were negative for MMR deficiency on the testing performed. For clinicians, this highlights the importance of early detection and emphasizes the utility of colonoscopic evaluation, given that it was the only method used that was able to identify and localize both masses.
